# Application of a preoperative pain management mode based on instant messaging software in elderly hip fracture patients: a randomized controlled trial

**DOI:** 10.1186/s12877-023-03905-2

**Published:** 2023-03-30

**Authors:** Yang Shen, Wei Liu, Zhe Zhu, Shuangmei Liu, Yanyan Cao, Lei Yan, Liang Chen

**Affiliations:** 1 Department of Emergency Medicine, Shengjing Hospital of China Medical University, No.36 Sanhao Street, Heping District, Shenyang, 110004 Liaoning Province China; 2 Department of Anesthesiology, Shengjing Hospital of China Medical University, No.36 Sanhao Street, Heping District, Shenyang, 110004 Liaoning Province China

**Keywords:** Hip fracture, Elderly adults, Pain management, Nerve block, Instant messaging software

## Abstract

**Background:**

Preoperative analgesia of hip fracture in elderly patients is important, but it is also lacking. In particular, nerve block was not provided in time. In order to provide more effective analgesia, we designed a multimodal pain management mode based on instant messaging software.

**Methods:**

From May to September 2022, a total of 100 patients with unilateral hip fracture aged over 65 were randomly divided into the test group and the control group. Finally, 44 patients in each group completed the result analysis. A new pain management mode was used in the test group. This mode focuses on the full information exchange between medical personnel in different departments, early fascia iliaca compartment block (FICB), and closed-loop pain management. Outcomes include the time when FICB is completed for the first time; The number of cases of FICB completed by emergency doctors; Patients' pain score, pain duration.

**Results:**

The time for patients in the test group to complete FICB for the first time was 3.0 [1.925–3.475] h, which was less than the time for patients in the control group (4.0 [3.300–5.275] h). The difference was statistically significant (*P* < 0.001). Compared with 16 patients in the control group, 24 patients in the test group completed FICB by emergency doctors, and there was no statistical difference between the two groups (*P* = 0.087). The test group was superior to the control group in the highest NRS score (4.00 [3.00–4.00] vs 5.00 [4.00–5.75]), the duration of the highest NRS score (20.00 [20.00–25.00] mins vs 40.00 [30.00–48.75] mins), and the NRS > 3 time (35.00 [20.00–45.00] mins vs 72.50 [60.00–45.00] mins). The analgesic satisfaction of patients in the test group (5.00 [4.00–5.00]) was also significantly higher than that of the control group (3.00 [3.00–4.00]). The above four indexes were different between the two groups (*P* < 0.001).

**Conclusions:**

Using instant messaging software, the new model of pain management can enable patients to receive FICB as soon as possible and improve the timeliness and effectiveness of analgesia.

**Trial registration:**

Chinese Clinical Registry Center, ChiCTR2200059013, 23/04/2022.

## Introduction

Hip fracture is a common fracture in elderly patients and is the main cause of serious morbidity in elderly individuals aged 65 years and above [1]. The direct costs associated with hip fracture are enormous and are accompanied by the development of other negative consequences, such as disability, depression and cardiovascular disease, at an additional cost to society [2]. The pain of hip fracture is severe, but it often does not receive enough analgesic treatment. Severe pain may induce cardiovascular and cerebrovascular accidents or other complications, which may affect the prognosis [3, 4]. Therefore, the reduction of postoperative complications should be considered in the perioperative period. Effective analgesia is a necessary means. Providing effective analgesia for patients has been proven to reduce the incidence of delirium, hospitalization time and hospital-acquired complications [5].

Pain management is particularly challenging in elderly patients in acute trauma settings [6]. Many countries have formulated guidelines to improve the treatment quality of elderly hip fracture patients [7, 8]. Perfect analgesia includes preoperative and postoperative analgesia, which will cover the whole process of patients after injury. The existing analgesic schemes always ignore preoperative analgesia [9]. Preoperative analgesia in the emergency department is limited by doctors' concepts and techniques, and there are still some concerning phenomena, such as untimely analgesic treatment and insufficient analgesic effects. In particular, the implementation of nerve block was still delayed.

At present, several studies have shown that the multidisciplinary cooperation analgesic mode has played a great role in the perioperative pain management of hip fractures [10, 11]. Interdisciplinary cooperation on hip fracture analgesia brings the whole treatment provider cycle of patients into a new path, creates a skilled medical care queue, minimizes obstacles, and can bring nerve block analgesia to more patients [12].

To improve the preoperative analgesia of elderly patients with hip fractures, we plan to implement an interdisciplinary analgesic mode. The purpose of the new mode is to advance the implementation time of nerve block analgesia and improve pain management by increasing communication between different departments. We designed a randomized controlled trial to determine the role of this new management method in the early analgesia of elderly hip fracture patients.

## Methods

### Study design and ethics

This study was designed as a prospective parallel-group randomized controlled trial and conducted at Shengjing Hospital. The investigation was approved by the Institutional Review Board of Shengjing Hospital, China Medical University (approval number 2021PS511K, dated 12/05/2021). The trial was registered with the Chinese Clinical Registry Center (registration No. ChiCTR2200059013, dated 23/04/2022) before patient enrolment. Written informed consent was obtained from all subjects participating in the trial. The trial protocol followed the Declaration of Helsinki. This manuscript adheres to the applicable Consolidated Standards of Reporting Trials (CONSORT) guidelines.

### Inclusion and exclusion criteria

Inclusion criteria: patients aged ≥ 65 years with unilateral hip fracture who were admitted to our emergency department and were willing to undergo surgical repair.

Exclusion criteria: multiple trauma or multiple fractures, coagulation disorders, gastrointestinal ulcers or bleeding, puncture site infections, history of allergy to local anaesthetics, opioid addiction, inability to communicate or score properly, presence of delirium, participation in other clinical trials.

### Randomization and blinding

In this parallel double-blind trial, the computer-generated random allocation sequence was created by an independent investigator using SPSS Statistics (version 24.0, IBM Corp., Armonk, NY, USA) with a 1:1 allocation and random block size. After providing written informed consent, the eligible patients were entered into the trial within 2 h of the emergency visit and were randomized into the trial and control groups using sealed opaque envelopes to reveal the treatment arm. None of the patients or data collectors were aware of the grouping.

### Interventions

#### Test group

The core aim of the new pain relief management mode is to establish a pain management group composed of medical personnel from the emergency department, orthopaedics department and anaesthesiology department. Members of the working group have received unified training on the new pain relief mode.

The communication within the group is through instant messaging software. All the accounts of the working group are public. The doctors on duty in each department swill log in to the account to join the working group during working hours and see past data and real-time information on their smartphones, computers and other devices. The data in the working group do not include private data other than the patient's medical information and will be completely cleared before the patient is discharged.

- 1. Admission evaluation: The emergency medical staff will conduct the first comprehensive evaluation after the patient is admitted to the hospital and will send the evaluation results (general information of the patient, trauma, numerical rating scale [NRS] score, coexisting diseases, history of past illness and allergy history) to the working group.
- 2. Analgesic scheme: All patients will be given a multimodal analgesic scheme before the operation. The basic analgesia is flurbiprofen axetil administered for 12 h. The affected side will be treated with 0.25% ropivacaine for fascia iliaca compartment block (FICB). The interval between two blocking operations should not be less than 8 h. After the completion of FICB, when the patient's pain NRS score is still > 3, he or she will take one tablet of oxycodone and acetaminophen orally.
- 3. FICB: FICB will be implemented as soon as conditions permit. FICB can be completed by any doctor familiar with the operation, such as an emergency department physician or anaesthesiologist. During the operation, the ultrasound images of patients will also be shared in the working group in real time, and other doctors familiar with the FICB operation will also conduct remote guidance and consultation.
- 4. Pain self-assessment: Educate patients on how to correctly use the NRS to assess their pain level. The NRS describes the pain intensity on a scale of 11 points that increase from 0 to 10. The specific scores are as follows: 0 indicates no pain, 1–3 indicates mild pain, 4–6 indicates moderate pain, 7–9 indicates severe pain, and 10 indicates the most severe pain. Patients with an NRS score of > 3 points can inform doctors to seek additional analgesic treatment.
- 5. Pain closed-loop management: The dosage and effect of FICB for each patient are used as a reference for continuous treatment, and the information is stored as a case in the shared document of the working group. The changes in patients' pain scores and the additional use of oral analgesics will be recorded and summarized, the changes in the patients' pain will be tracked, and these data will be published to the working group. For patients with high pain scores, intragroup consultation will be conducted to consider increasing the concentration or dose of drugs in the next FICB.

#### Control group

In this study, each patient's analgesia was directed by the doctor in his or her department, and other departments were invited to consult if necessary.

- 1. Admission evaluation: The emergency medical staff carried out the first comprehensive evaluation after the patient was admitted to the hospital, and the contents were the same as those of the test group.
- 2. Analgesic scheme: This scheme was the same as that in the test group.
- 3. FICB: This was performed when the emergency or orthopaedic doctor considered it necessary or had asked the anaesthesiologist for a consultation.
- 4. Pain self-assessment: This was consistent with the guidance provided to the test group.

After participating in the trial, those patients who entered the intensive care unit preoperatively were excluded. Patients who ultimately did not undergo surgery were excluded from the trial. Patients with unplanned surgery during this hospitalization, that is, any surgery except for a single hip fracture surgery (including internal fixation and total/half hip replacement), were excluded.

The anaesthesia method was not limited during the operation, and the postoperative analgesia scheme comprised a single FICB and an intravenous analgesia pump containing sufentanil.

### Outcome measurements

- 1. Time from the emergency room visit to the completion of FICB.
- 2. Number of FICB completed by emergency physicians.
- 3. The maximum NRS, the duration of the maximum NRS, and the duration of NRS > 3 were recorded.
- 4. Analgesic satisfaction score. Patients were given a satisfaction score for preoperative analgesia before the procedure using a 5-point Likert scale from 1 to 5, ranging from complete disagreement to complete agreement, with higher scores indicating greater satisfaction with pain control.
- 5. Incidence of postoperative delirium (POD) in the first 7 days postoperatively. Daily Confusion Assessment Method (CAM) scores were assessed by a blinded assessor in a face-to-face assessment [13].

### Statistical analysis

#### Sample size calculations

The main aim of this study was to compare the first FICB times. Based on the results of the pretest, we assumed that the first FICB timepoint for the test group was 3.0 h and that for the control group was 4.0 h, both with a standard deviation of 1.5. To obtain a statistical power of 90% (β = 0.1) with a two-sided confidence interval of 95% (α = 0.05), we used PASS11 for calculations with a sample size of 80 patients (40 in each group). The study planned to recruit an additional 10% of patients to account for potential loss to follow-up and withdrawals.

#### Statistical methods

SPSS 24 software was used for statistical analysis. The distribution of continuous variables was tested for normality using Shapiro‒Wilk tests. Normally distributed data were expressed as the mean ± standard deviation (SD), while nonnormally distributed data were expressed as the median (interquartile range [IQR]). Count data were expressed as numbers. The between-group comparison was performed using an independent t test for normally distributed measurement data and the Mann‒Whitney U test for nonnormally distributed measurement data. Count data were compared using a Pearson chi-square test or Fisher’s exact test. The data were compared using a Pearson chi-square test or Fisher’s exact test.

Intention to treat (ITT) was used in this study. Because in the actual clinical work, the medical staff treating the patients in the control group may have received training on the new mode. Inevitably, the medical staff cannot completely reject the new mode in the process of treatment. Therefore, we will use ITT, that is, analysis based on intention to treat (i.e., planned grouping), rather than actual treatment.

## Results

**Fig. 1:**
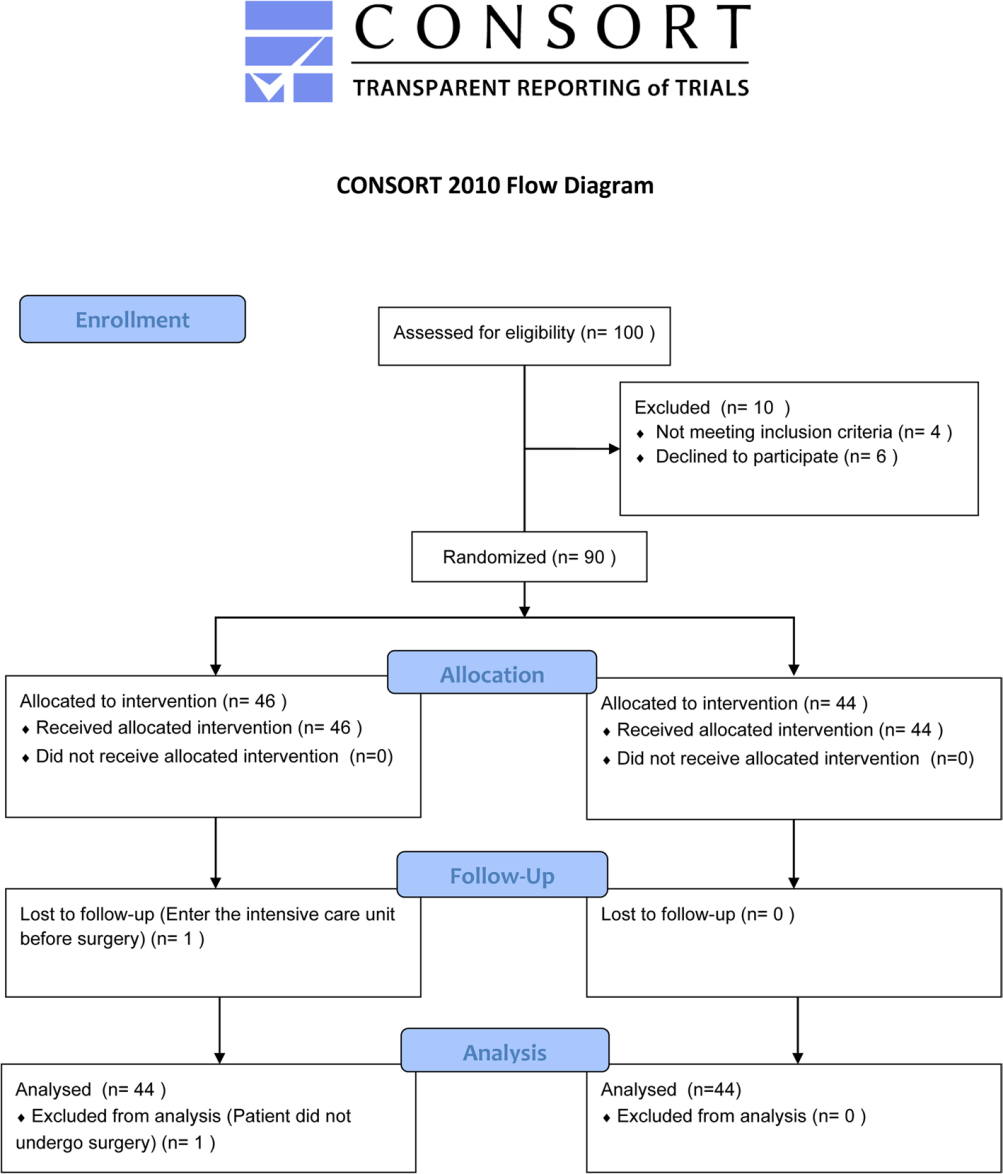
CONSORT flow diagram

A total of 100 patients were enrolled in the study from May to September 2022, with 88 patients eventually completing the primary outcome analysis (see Fig. 1). There were no significant differences between the two groups in terms of age, sex composition ratio, height, weight, BMI or time from admission to surgery. There was no difference in the level of education and Mini-Mental State Exam (MMSE) score between the two groups. See Table 1 for details.

**Table 1:** Comparison of preoperative information between two groups

	Test group (*n* = 44)	Control group (*n* = 44)	test value	*P* value
Age (years)	77.6 ± 7.3	76.6 ± 6.1	0.699	0.487
Sex (male/female)	18/26	16/28	0.192	0.661
Height (cm)	165.3 ± 5.0	165.0 ± 5.2	0.230	0.819
Body weight (kg)	59.8 ± 6.1	58.0 ± 6.2	1.419	0.159
BMI	21.9 ± 1.9	21.2 ± 1.5	1.772	0.080
Time from admission to operation (hours)	34.5 [26.0–40.75]	34.5 [28.25–44.75]	867.500	0.401
Level of education			0.090	0.764
< Elementary school	0	0		
Elementary school	6	7		
≥ Secondary school	38	37		
MMSE	27.00 [25.00–29.75]	27.00 [25.00–28.75]	858.500	0.355

Data are expressed as the mean ± standard deviation (SD), median (IQR) or numbers

*BMI* Body mass index, *MMSE* Mini-Mental State Exam

The time to first FICB completion for patients in the test group was 3.0 [1.925–3.475] h, which was less than the time to first FICB completion for patients in the control group, which was 4.0 [3.300–5.275] h. The difference between the two groups was statistically significant (*P* < 0.001). Relative to the 16 patients in the control group, 24 patients in the trial group had their FICB operation completed by an emergency physician, and although the absolute value was higher in the trial group than in the control group, there was no significant difference between the two groups (*P* = 0.087). No patients in either group developed complications arising from the FICB operation.

In comparing pain-related indicators between the two groups, the test group outperformed the control group in terms of the maximum NRS, the duration of the maximum NRS, and the duration of NRS > 3. The patients in the trial group also had significantly higher analgesic satisfaction than the control group. See Table 2.

**Table 2:** Pain score, duration and analgesic satisfaction score of patients

	Test group (*n* = 44)	Control group (*n* = 44)	Test value	*P* value
Maximum NRS	4.00 [3.00–4.00]	5.00 [4.00–5.75]	192.000	< 0.001
Duration of the maximum NRS (mins)	20.00 [20.00–25.00]	40.00 [30.00–48.75]	187.000	< 0.001
Duration of NRS > 3 (mins)	35.00 [20.00–45.00]	72.50 [60.00–45.00]	26.000	< 0.001
Analgesic satisfaction score	5.00 [4.00–5.00]	3.00 [3.00–4.00]	180.500	< 0.001

Data are expressed as the median (IQR)

Within 7 days postoperatively, two patients in the trial group developed POD, and four patients in the control group developed POD, with no difference between the two groups (Fisher's exact probability 0.715, *P* = 0.338).

## Discussion

The results of this study showed that compared with the control group, the patients in the test group received FICB earlier, and their satisfaction with analgesia was improved. The maximum NRS of patients in the test group was lower, and the duration of the maximum NRS and duration of NRS > 3 was shorter.

It has been widely recognized that the perioperative management of hip fractures, especially pain management, requires multidisciplinary cooperation. In this study, the results of the new mode of pain management were better than those of the control group. The primary reason for this is multidisciplinary cooperation. Multidisciplinary collaborative management for elderly hip fracture patients should involve the participation of emergency doctors, anaesthesiologists and orthopaedic doctors. Its contents include standardized first-aid procedures, comprehensive analgesic programmes, preoperative evaluation and treatment, early surgery and early discharge [11]. Multidisciplinary cooperation together forms a skilled doctor cohort, bringing the entire care of hip fracture patients into one treatment path [12]. In the new mode adopted by the trial group, anaesthesiologists participated in the patient management at an early stage to help complete FICB as soon as possible. Similar schemes have been proposed in previous studies. Anaesthesiologists participate in patient management, including analgesia, fluid resuscitation and standardized preoperative evaluation, after patients are admitted to the hospital [10].

Sufficient information exchange and feedback between different departments is the core of the new mode. With the progress of information science and technology, medical informatization has gradually become an important development trend. As a widely used information medium, instant messaging software provides users with more rapid, convenient and comprehensive cross-platform support. It has the characteristics of interactivity and immediacy. Using the universality of the network to promote the exchange and transmission of information, such software can meet the needs of users for instant information, provide a communication platform for team information communication, and make people's communication no longer limited by time, geographical location and space.

With the help of instant messaging software, medical personnel participating in preoperative pain management can share information, communicate fully, give feedback in a timely manner and make common progress. The timeliness of information exchange makes the formulation and adjustment of analgesic programme more efficient and effective. The individualized analgesic programme can improve the level of pain management and patient satisfaction. Especially in FICB operation, the operator can live broadcast the real-time picture of puncture in software video, including the operator's specific actions and ultrasound images. In cases of a difficult procedure where the operator is not skilled enough, other doctors can remotely guide the operator to complete the block in real time to improve the success rate. In this study, the experimental group completed FICB earlier, which is precisely because of this approach.

At the same time, under the new mode, medical staff are more familiar with patient information, especially when transferring them from the emergency department to the orthopaedics department. Medical staff have enough knowledge of the general situation and analgesia status of the patients, which will make patients feel more at ease and increase their overall satisfaction.

The new mode in this study showed a better analgesic effect, which was directly due to the early implementation of FICB.

A nerve block is an important part of the treatment plan for hip fracture patients and a key factor for success. Many previous studies have confirmed that single nerve block, especially FICB technology, has shown good analgesic effects in the preoperative analgesia of hip fracture in prehospital emergency settings and in emergency rooms [14–16]. FICB can block the femoral nerve, lateral femoral cutaneous nerve and obturator nerve, effectively alleviating the pain of hip fracture. Its operating position is supine, which can be safely and quickly used in an acute environment under the guidance of ultrasound. It is implemented by trained medical personnel, and the effect is good. The advantage of FICB is that it can reduce the use of opioids and prolong the time of first use of opioids while effectively relieving pain. Reducing the risk of pulmonary complications, reducing the cost of analgesia, and reducing postoperative cognitive dysfunction may further improve the incidence rate, mortality and quality of life [14, 17]. Early FICB and multidisciplinary cooperation decreased the maximum NRS score of patients and lasted for a short time. The duration of NRS > 3 was also shortened. Timely and effective analgesia improved the satisfaction of patients.

Adequate analgesia can reduce the incidence of POD in elderly patients. POD is an important factor affecting the prognosis of elderly patients. POD is an acute and reversible mental disorder characterized by changes in the level of consciousness and attention disorders after surgery and is a common postoperative complication of the central nervous system in elderly patients [18]. POD can cause long-term cognitive impairment and physical function decline in patients, leading to prolonged hospitalization and even affecting long-term outcomes. Studies have shown that 10%—16% of emergency hip fracture patients may have delirium [19]. Delirium is an independent risk factor for death, institutionalization and dementia, and providing effective analgesia to patients has been proven to reduce delirium [20–22]. The incidence of POD after surgery in elderly hip fracture patients is even higher [23]. Many studies on postoperative analgesia of hip fractures have confirmed that nerve block analgesia can prevent delirium [5, 14, 24]. The influence of nerve block on delirium may be multifactorial, including improving pain, enhancing analgesic effects and reducing opioid consumption [25]. However, preoperative analgesia for elderly hip fracture patients is missing in medical care. Twenty-nine percent of patients have no analgesic record in the emergency room, and only 7% of patients receive nerve block analgesia [9].

This study attempted to determine the preventive effect of preoperative analgesia on POD. The MMSE scale was used to compare the cognitive function of the two groups of patients when they were admitted to the hospital because cognitive function change is an independent risk factor for delirium after orthopaedic surgery in the elderly [26]. There was no difference between the two groups. There were no restrictions on the anaesthesia mode during the operation because studies have shown that the anaesthesia mode does not affect the incidence of POD [27]. However, the results of this study showed that although the number of POD cases in the test group was less than that in the control group, there was no significant difference in the incidence of POD between the two groups. This may have been caused by the small sample size.

### Limitations

- 1. There was no difference in the results of POD in this study. A large sample size will be used in future studies to determine the effect of preoperative analgesia on POD.
- 2. In addition to POD, this study did not analyze the impact of prognostic indicators of patients under the new model. We look forward to finding the possible influence of analgesia mode on prognosis in future research.
- 3. In the new mode, instant messaging software is used to enhance the convenience of information exchange, and attention should be given to protecting patient privacy and information security during use. We hope there will be specialized software in the future.

## Conclusions

For elderly patients with hip fracture, the new pain management mode, supported by instant messaging software, given full play to multidisciplinary cooperation and information exchange to complete FICB as soon as possible for efficient and effective analgesic treatment of patients.

## Data Availability

The research data has been uploaded to the database. The public access to the database is open. https://doi.org/10.6084/m9.figshare.21086659. The datasets used and/or analysed during the current study are available from the corresponding author on reasonable request.

## Abbreviations

CONSORT: Consolidated Standards of Reporting Trials
FICB: Fascia iliaca compartment block
POD: Postoperative delirium
CAM: Confusion assessment method
SD: Standard deviation
IQR: Interquartile range
ITT: Intention to treat
MMSE: Mini-Mental State Exam
